# Fast, Resource-Saving, and Anti-Collaborative Attack Trust Computing Scheme Based on Cross-Validation for Clustered Wireless Sensor Networks

**DOI:** 10.3390/s20061592

**Published:** 2020-03-12

**Authors:** Chuanyi Liu, Xiaoyong Li

**Affiliations:** 1 Harbin Institute of Technology (Shenzhen), School of Computer, Shenzhen 518055, China; liuchuanyi@hit.edu.cn; 2 Cyberspace Security Research Center, Peng Cheng Laboratory, Shenzhen 518000, China; 3 The Key Laboratory of Trustworthy Distributed Computing and Service, Ministry of Education, Beijing University of Posts and Telecommunications, Beijing 100876, China

**Keywords:** cross-validation, anti-collaborative attack, resource-saving, trust computing, wireless sensor networks

## Abstract

The trust computing mechanism has an increasing role in the cooperative work of wireless sensor networks. However, the computing speed, resource overhead, and anti-collaborative attack ability of a trust mechanism itself are three key challenging issues for any open and resource-constrained wireless sensor networks. In this study, we propose a fast, resource-saving, and anti-collaborative attack trust computing scheme (FRAT) based on across-validation mechanism for clustered wireless sensor networks. First, according to the inherent relationship among three network entities (which are made up of three types of network nodes, namely base stations, cluster heads, and cluster members), we propose the cross-validation mechanism, which is effective and reliable against collaborative attacks caused by malicious nodes. Then, we adopt a fast and resource-saving trust computing scheme for cooperation between between cluster heads or cluster members. This scheme is suitable for wireless sensor networks because it facilitates resource-saving. Through theoretical analysis and experiments, the feasibility and effectiveness of the trust computing scheme proposed in this study are verified.

## 1. Introduction

Wireless sensor networks (WSNs [1,2,3,4,5]) are widely used in several fields such as intelligent perception, military, disaster warning, medical care, etc. The main application of WSN is to sense the surrounding environment and send the obtained information to the base station (BS) for subsequent processing. For clustered WSNs such as EEHC [6], EC [7], HEED [8], TRAST [9], and LDTS [10], clustering algorithms can significantly improve the performance and efficiency of wireless sensor networks [11]. The clustering algorithm is used to divide nodes into multiple clusters. In each cluster, a node with powerful computing capability is selected as the cluster head (CH). Multiple CHs together form a higher level information transmission network. This layered network structure helps increase the speed of data collection and can limit network operations that consume large amounts of bandwidth [10,12]. Many applications in WSNs require coordination through wireless communications between participating nodes for interactive operations such as task collaborations and data transmissions [10,13,14,15].

However, the inherent security issues of WSNs also arise in the cooperation between participating nodes. WSNs are usually highly accessible in existing applications, which makes them very vulnerable to malicious attacks. Therefore, it becomes very important to provide a secure and trusted collaboration mechanism for WSNs. The trust mechanism and network entity behavior are an important factors that WSNs must consider [10,16,17,18,19,20]. The trust mechanism can be used to detect the reliability and security of the cooperative nodes (or to identify the faulty nodes) or to assist in the decision-making process, such as whether a node needs to choose a partner to complete the data transmission task [21,22,23,24,25,26].

### 1.1. Challenges and Motivations of This Work

The trust mechanism is an important element in any network computing environment [27,28,29,30,31,32,33]. There are many advantages to introducing a trust mechanism in clustered WSNs [16,17,18,19,20] and selecting a CH to detect failed or malicious nodes in the cluster [34]. In a multi-hop cluster environment [8], the trust mechanism supports the selection of a trusted routing node (usually a CH), and a cluster member (CM) can send the collected data to the CH. In communication between clusters, the trust mechanism also supports the selection of trustworthy routing gateway nodes or other trustworthy CHs through which the sender forwards data to the BS [10]. The BS is a powerful device that can process the information collected from the CM and interact with the user.

However, due to its high resource consumption (such as memory, time, and communication overhead), this makes traditional trust computing solutions developed for wired and wireless ad hoc networks unsuitable for sensor networks. The computing speed and resource-saving problem of a trust system are the most key requirements for resource-constrained WSNs. At the same time, the anti-collaborative attack ability of a trust computing mechanism itself is another challenging issue for any WSN (including clustered WSNs). Currently, there is a lack of a universal trust computing solution designed for clustered WSNs that can simultaneously achieve computing speed, resource efficiency, and resistance to collaborative attacks.

- *Most studies do not consider both computational speed and resource overhead issues of the trust computing scheme itself.* The trust mechanism should be fast and save resources to serve a large number of resource-constrained nodes in terms of accuracy, calculation speed, storage overhead, and communication overhead [17,18,35]. Currently, many representative works have been proposed for clustering WSNs, such as the group-based trust computing mechanism (GTMS) [18], the belief-based trust evaluation mechanism (BTEM) [34], the trust and reputation scheme (ATRM) [36], the trust-based cluster head election mechanism (TCHEM) [37]. However, most of these studies do not simultaneously consider the computational speed and resource overhead issues of the trust computing scheme itself. Most of these studies use complex trust calculation algorithms at each CM or CH, which will greatly affect the applicability of the trust model.
- *Most studies do not consider the anti-collaborative attack ability of the trust computing scheme itself.* The malicious nodes may cooperate to provide false feedback information to attack the trust computing system. The anti-collaborative attack ability of the trust computing scheme should have the ability to identify cooperative attacks. WSNs are usually deployed in insecure and highly complex network environments, and nodes may be attacked and cooperatively spoofed. Furthermore, an attacker can disrupt communication or spread misleading sensor values through sensor nodes that have been compromised. In the traditional WSN trust mechanism, the trust system collects remote feedback and then aggregates such feedback to generate a global trust for the node, which can be used to evaluate the overall trust of the node (such as TCHEM [37] and HTMP [34]). There are a large number of malicious nodes in an open or hostile WSN environment. The ratings of these malicious nodes may produce erroneous results. Organized cooperative attacks are the most important threat to trust mechanisms in WSNs [10,12,18]. However, most previous studies lacked consideration of malicious cooperative attacks, which severely affected the security, availability, and reliability of the system.

By identifying the inherent relationship among CMs, CHs, and BSs, we propose a fast, resource-saving, and anti-collaborative attack trust computing scheme based on the cross-validation mechanism. This scheme can effectively eliminate collaborative attacks initiated by large collaborative groups and large-scale malicious nodes. Different from the previous trust computing methods, in the proposed trust mechanism, feedback comes not only from CMs, but also from CHs and BSs. This cross-validation mechanism can effectively reduce malicious feedback and improve system security.

### 1.2. Main Idea and Contributions

To the best of our knowledge, this study is the first to construct a fast, resource-saving, and anti-collaborative attack trust computing scheme based on an innovative cross-validation mechanism. Compared with existing methods, the main contributions of this paper are as follows:

- A cross-validation trust aggregating mechanism, which has anti-collaborative attack ability against garnished and collaborative attacks caused by malicious nodes. In the proposed cross-validation trust aggregating mechanism, feedback not only comes from CMs, but also from CHs and BSs. The feedback information from multiple sources confirms each and constitutes a cross-validation mechanism. Such CM-level trust computing, with three trust factors, including CM-to-CM direct trust, CM-to-CM feedback, and CH-to-CM feedback, constitutes a cross-validation relationship. For CH-level trust computing, three trust factors, CH-to-CH direct trust, CH-to-CH feedback, and BS-to-CH feedback, also constitute a cross-validation relationship. This cross-validation mechanism can effectively reduce the risk of the system, while improving system reliability and security. We investigated representative trust schemes in clustered WSNs, such as LDTS [10], GTMS [18], DST [19], BTEM [34], ATRM [36], and TCHEM [37]. We found that many of these studies lacked considerations of the anti-collaborative attack ability of the trust scheme itself. We extended the traditional trust schemes in clustered WSNs and proposed a cross-validation trust mechanism based on multiple trust factors, which has a stronger anti-collaborative attack ability against collaborative attacks compared with existing trust mechanisms.
- A fast and resource-saving trust computing scheme for cooperation between CMs or between CHs, which is suitable for resource-constrained WSNs. The computational speed and resource-saving of a trust system are the most fundamental requirements for resource-constrained WSNs. However, most of these studies (such as LDTS [10], GTMS [18], DST [19], BTEM [34], ATRM [36], and TCHEM [37]) failed to consider the resource efficiency issue of the trust computing scheme itself. In this study, the number of successful transmissions was considered as the key credential to determine the trustworthiness of a node. We adopted fast algorithms and a resource-saving mechanism to compute the trust value between nodes, which was suitable for resource-constrained WSNs with large-scale nodes.

Together, these innovative designs made the fast, resource-saving, and anti-collaborative attack trust computing scheme (FRAT) solution a fast, resource-efficient, and cooperative attack-resistant solution that could be used in a clustered WSN environment. This study provided the theoretical basis and experimental results for verifying the design of FRAT. Theoretical analysis and experimental results showed that compared with the existing methods, FRAT had superior performance.

The main contents of the rest of this study are as follows: Section 2 provides an overview of related work. The cross-validation mechanism for trust computing for clustered WSNs is described in Section 3. Section 4 gives the details of the trust scheme in the FRAT scheme. Section 5 and Section 6 respectively provide the theoretical and experimental analyses of FRAT. Section 7 is the conclusion of this paper.

## 2. Related Work

Desai et al. proposed a trust evaluation method that used node’s internal resources to evaluate node-level trust [12]. Using the suggested self-test algorithm, this method helped nodes trust themselves after booting by ensuring reliable system memory. This algorithm was a completely intermediary technology and had nothing to do with the network topology and auxiliary information.

In [18], a group-based trust computing mechanism (GTMS) was proposed for clustered WSNs. Compared with traditional trust schemes that always focus on the trust value of a single node, GTMS evaluates the trust of a group of nodes. This approach provides a benefit to WSNs, which requires less memory to store trust records on each node. GTMS helps to reduce the costs associated with trust evaluation of remote nodes significantly.

In [34], a belief-based trust evaluation mechanism (BTEM) was developed for wireless sensor networks. The proposed mechanism could resist against various network attacks such as on-off attacks, bad-mouth attacks, and DoS (denial of service) attacks. Simulation-based experimental results showed that the trust mechanism could not only successfully identify and isolate malicious nodes to a certain extent, but also improve the detection rate of malicious behaviors.

In [37], a trust-based cluster head (CH) election mechanism (TCHEM) was proposed. Its basic framework was proposed based on the clustered network model. In this network collaboration model, all nodes had unique local IDs. This method could reduce the possibility of malicious or damaged nodes becoming CHs. This mechanism discouraged sharing trust information between sensor nodes. Therefore, this method reduced the impact of cooperative attacks.

In [36], a trust and reputation scheme (ATRM) based on a distributed agent mechanism was proposed for WSNs. With the help of a mobile agent running on each network node, ATRM collects trusted information and calculates the node’s trust. The benefits of local management schemes for trust and reputation are that there is no need for a centralized repository, and the node itself can provide its own reputation information when needed. As a result, there are no network-wide floods or acquisition delays when performing reputation calculations and propagation.

In [35], the authors proposed a robust trust-aware routing framework (TARF) for dynamic WSNs. Because there was no consideration of tight time synchronization, TARF provided a reliable and energy-saving trust scheme. Facts have proven that TARF could effectively prevent harmful attacks due to identity spoofing; through simulation and empirical experiments on large WSNs in various scenarios including mobile and RF shielded network conditions, verifying the flexibility of TARF through extensive evaluation. In addition, the authors implemented a low-overhead TARF module. This implementation could be incorporated into existing routing protocols with minimal changes.

In [10], the authors proposed LDTS, a lightweight and highly reliable trust system for collecting data through wireless sensor networks. First, a lightweight trust decision scheme based on node identity (role) in a clustered wireless sensor network was proposed. This scheme is suitable for such wireless sensor networks because it is conducive to energy saving. Because feedback between cluster members (CM) or the cluster head (CH) is eliminated, this method can greatly improve system efficiency while reducing attacks on the system by malicious nodes. Considering that the CH undertook a large number of data forwarding and communication tasks, this study defined the cooperation of reliability enhancement trust assessment methods between CHs. This method could effectively reduce the network consumption caused by malicious or selfish CHs.

The research on the trust mechanism for WSNs has received extensive attention from scholars. In WSNs, how to identify malicious nodes accurately is a challenging problem that has aroused widespread concern in academia and industry. Table 1 concludes about the features of the trust computing mechanism for WSNs mentioned. From Table 1, we can find that, in view of the security and trustworthiness of WSNs, some feasible and rich solutions were proposed [10,12,18,34,35,36,37], but an efficient trust computing mechanism designed for clustered WSNs from the simultaneous achievement of overhead-saving and anti-collaborative attack ability is still necessary.

## 3. Cross-Validation Mechanism for Trust Computing

In this section, we first present the conceptual model and formal definitions based on the cross-validation mechanism, which is employed by FRAT. We then establish a trustworthy clustered WSN environment based on the trust relationship among network entities (CMs, CHs, and BSs). We will also analyze possible attack patterns that threaten to build trust relationships.

### 3.1. Three-Tier Network Architecture Model

The clustering algorithm provides one of the most feasible solutions for communication in WSNs due to its inherent resource-saving characteristics and its suitability for highly scalable networks. The FRAT solution is based on a clustered WSN with a backbone, and its core function is to build a reliable and efficient data aggregation network.

**Figure 1:**
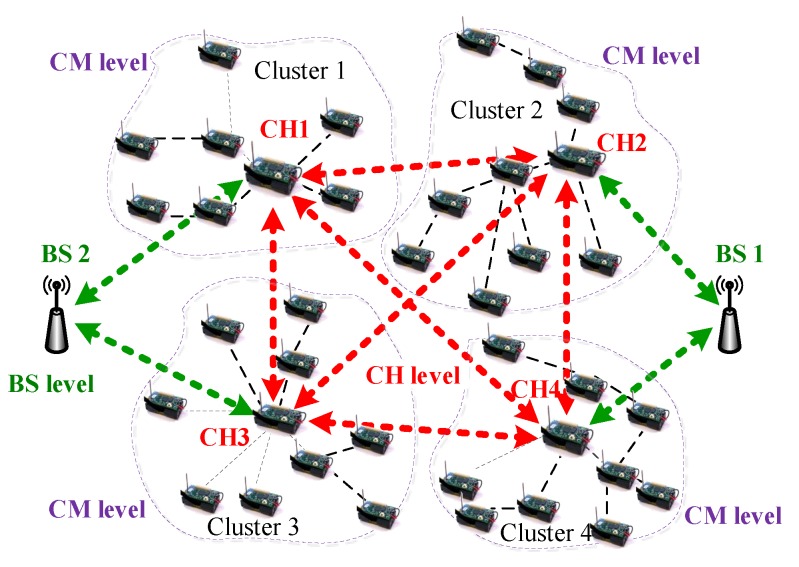
Three-tier network architecture model, in which a member can be identified as a BS, a CH, or a CM according to their features.

As shown in Figure 1, according to its characteristics, the nodes in a clustered WSN environment can be identified as the CH or CM [10]. The CM in the cluster can communicate directly with its CH. The communication between the CM and the BS can only be performed through the CH. In each cluster, only the CH can forward data directly to the BS. The CH collects, aggregates, and forwards data from the CM to the BS. The BS, CH and CM form a three-layer network architecture model (Figure 1).

One of the key tasks of this study is to construct a trust-based network topology model that can reduce the possibility of malicious members being selected as cooperative partners in data forwarding. Through the cooperation of other CHs, one CH can forward the collected data to the central BS node. It was assumed that the members were divided into multiple clusters based on existing clustering algorithms, such as [8,10]. We also assumed that each node had a unique ID, which could be used to distinguish it from other nodes, similar to the assumptions in [18,34,37]. Once the cluster was formed, they would maintain the same CMs unless a CM was blacklisted or dead or a new node joined the sensor network.

### 3.2. Formal Definitions of Trust Based on Cross-Validation

**Figure 2:**
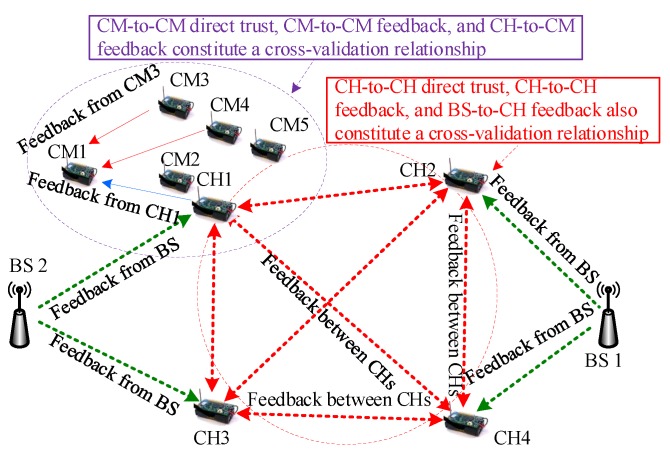
Cross-validation mechanism for trust computing based on the three-tier network architecture model.

Based on the inherent relationship among CMs, CHs, and BSs, this paper first systematically studies and constructs a cross-validation trust computing scheme for clustered WSN environment. In Figure 2, following the functions of the network nodes in the cluster WSN, a total of three network entities exists, namely CMs, CHs, and BSs. Thus, a total of three collaborative groups can be formed: a CM group ($\left\{CM_{1},CM_{2},\cdots ,CM_{i},\cdots ,CM_{I}\right\}$, where *i* is the unique identity of a CM, *I* is the total number of CM nodes in the system); a CH group ($\left\{CH_{1},CH_{2},\cdots ,CH_{j},\cdots ,CH_{J}\right\}$, where *j* is the unique identity of a CH, *J* is the total number of CHs); and a BS group ($\left\{BS_{1},BS_{2},\cdots ,BS_{k},\cdots ,BS_{K}\right\}$, where *k* is the unique identity of a BS, *K* is the total number of BSs). There are two basic trust relationships between these network entities. One is the trust relationship between two CMs. This is the most basic trust relationship in a clustered WSN environment [12]. The other is the trust relationship between two CHs, and this is a special trust relationship in the clustered wireless sensor network environment. This is a crucial factor in encouraging cooperation between CHs and is highly important in successfully deploying a trustworthy clustered WSN. Referring to the methods in [10,33,38], we then provide the cross-validation definitions of the trust relationship used in the clustered WSN environment.

The main innovation of the cross-validation approach is embodied in the following two aspects:

- First, three trust factors constitute a cross-validation relationship in the CM-level (or CH-level) trust computing. For CM-level trust computing, three trust factors, including CM-to-CM direct trust, CM-to-CM feedback, and CH-to-CM feedback, constitute a cross-validation relationship. For CH-level trust computing, three trust factors, CH-to-CH direct trust, CH-to-CH feedback, and BS-to-CH feedback, also constitute a cross-validation relationship. Different from the previous trust computing methods, in the proposed trust mechanism, feedback not only comes from CMs, but also from CHs and BSs. This cross-validation mechanism can effectively reduce the risk of the system, while improving system reliability and security.
- Second, relying on the theory of standard deviation analysis [39,40], we used an aggregating method for the overall trust degree in which three trust factors were further cross-validated with one another. In statistics, deviation analysis refers to the absolute difference between any number in a set and the mean of the set [40]. Different from traditional methods, our mechanism based on the theory of deviation analysis is a cross-validated trust calculation mechanism based on multiple trust factors (in CM-level trust computing, including CM-to-CM direct trust, CM-to-CM feedback, and CH-to-CM feedback; in CH-level trust computing, including CH-to-CH direct trust, CH-to-CH feedback, and BS-to-CH feedback). The trust factor with a larger deviation compared with the other two values is eliminated from the overall trust aggregation process. At the same time, this removal solves the adaptive aggregation problem caused by malicious nodes (malicious CMs or CHs).

**Definition** **1.** ***Trust relationship between two CMs based on the cross-validation mechanism (called CM-to-CM overall trust).** The CM-to-CM overall trust is a quantifiable value of the competence of another CM (the CM to be evaluated) to complete the task of the CM, based on the CM’s direct evaluation and the feedback of CHs and other CMs. As the CH feedback information is integrated into CM-to-CM overall trust computing, this CM-to-CM overall trust computing approach is a cross-validation mechanism.*

Definition 1 and Figure 2 shows that the overall trust degree from CM to CM is the result of fusion calculation through three trust factors, namely CM-to-CM direct evaluation, CH-to-CM feedback, and CM-to-CM feedback. Due to feedback from two sources, this trust computing approach is called the cross-validation mechanism. In traditional feedback-based trust calculation mechanisms, such as in [12], feedback information mainly comes from the CMs, which could cause many problems, such as malicious attacks and coordinated deception. In a clustered WSN, a CH is usually selected by CMs according to its reliability, such as power, data forwarding success rate, and trust. Thus, feedback from the CHs should have higher reliability. From this point of view, the cross-validation mechanism can minimize system risks and improve the security of clustered WSNs.

**Definition** **2.** ***Trust relationship between two CHs based on the cross-validation mechanism (called CH-to-CH overall trust).** The CH-to-CH overall trust is a a quantifiable value in the judgment of another CH (the CH to be evaluated) to complete the task of the CH, based on the CH’s direct evaluation, and the feedback of BSs and other CHs (as the BS feedback information is integrated into CH-to-CH overall trust calculation, this CH-to-CH overall trust computing approach is a cross-validation mechanism).*

Similar to CM-to-CM overall trust, the overall trust of CH-to-CH is the result of aggregation calculation through three trust factors, namely CH-to-CH direct evaluation, CH-to-CH feedback, and BS-to-CH feedback. To integrate more reliable feedback from BSs, existing BS equipment is usually managed by a reputable ISP. The CH-to-CH overall trust is significantly enhanced. In addition, the basic function of BSs allows for dynamic monitoring of the forwarding behavior of CHs. Thus, each BS could provide feedback based on real monitoring data, which could then partly solve the problem of malicious feedback from CHs.

**Definition** **3.** ***Feedback between two CMs or between two CHs (called CM-to-CM feedback or CH-to-CH feedback).** Feedback between two CMs (or between two CHs) is a rating based on the CM or CH history behavior. After the data forwarding task is completed, the CM or CH will calculate the real-time trust. When another CM or CH requests it, the CH provides the value to the requester.*

**Definition** **4.** ***Feedback of a CH to a CM (called CH-to-CM feedback).** CH’s feedback on the CM is an objective rating based on the historical behavior of the CM. A CH dynamically monitors the CM behavior during the data forwarding. After the data forwarding task is completed, the CH calculates the real-time trust of the CM. When another CM requests it, the CH provides the value to the requester.*

**Definition** **5.** ***Feedback of a BS to a CH (called BS-to-CH feedback).** BS’s feedback on the CH is an objective evaluation based on the historical behavior of the CH. A BS dynamically monitors the CH behavior during the data forwarding. After the data forwarding task is completed, the BS will calculate the real-time trust of the CH. When another CH requests it, the BS provides the value to the requester.*

According to Definitions 1 and 2, the FRAT scheme needs to maintain two levels of trust relationship: CM-to-CM overall trust and *CH-to-CH* overall trust. In this paper, CM-to-CM overall trust is represented by $T_{CM_{x},CM_{y}}\left(\Delta t\right)$, and CH-to-CH overall trust is represented by $T_{CH_{i},CH_{j}}\left(\Delta t\right)$. Since trust is a dynamic value that changes over time, we added a timestamp Deltat to the expression. Likewise, the FRAT scheme needs to maintain four levels of feedback relationship: (1) feedback between a CM and a CM, (2) feedback of a CH to a CM, (3) feedback of a CH to a CH, and (4) feedback of a BS to a CH. We use $F_{CM_{x},CM_{y}}\left(\Delta t\right)$ to represent CM-to-CM feedback (use $F_{CH_{i},CH_{j}}\left(\Delta t\right)$ to represent *CH-to-CH* feedback), $F_{CH_{i},CM_{x}}\left(\Delta t\right)$ to represent *CH-to-CH* feedback, $F_{CH_{i},CH_{j}}\left(\Delta t\right)$ to represent *CH-to-CH* feedback, and $F_{BS_{z},CH_{j}}\left(\Delta t\right)$ to represent *BS-to-CH* feedback. $D_{CM_{x},CM_{y}}\left(\Delta t\right)$ and $D_{CH_{i},CH_{j}}\left(\Delta t\right)$ are the direct trust between two CMs or CHs. These are different from traditional trust computing methods (such as LDTS [10], GTMS [18], DST [19], BTEM [34], ATRM [36], and TCHEM [37]), in which the feedback comes from a single source. In summary, in the proposed FRAT scheme, the feedback information comes from multiple mutual cross-validation sources. Three types of feedback relationship form a cross-validation mechanism, and this mechanism has a protective ability against collaborative attacks caused by malicious nodes through the theory of deviation analysis.

**Figure 3:**
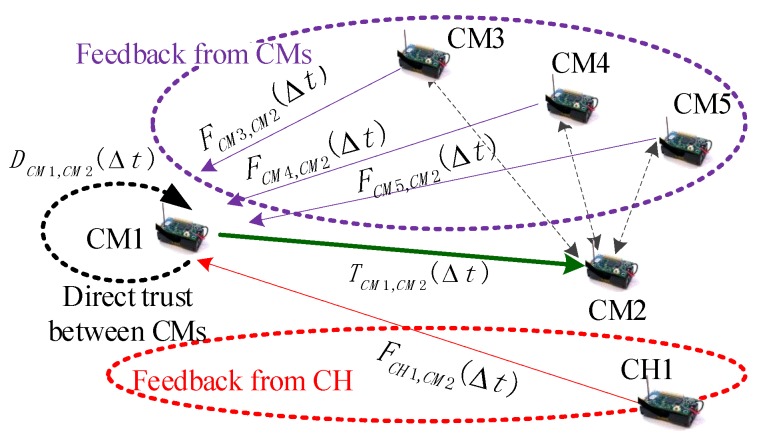
CM-to-CM overall trust computing based on the cross-validation mechanism. CM-to-CM direct trust, CM-to-CM feedback, and CH-to-CM feedback constitute a cross-validation relationship.

To clarify the cross-validation mechanism, we provide the following example. Consider the case in Figure 3, where CM-to-CM overall trust is computed based on the cross-validation mechanism. In this case, if CM1 wants to compute the overall trust of CM2, CM1 first asks for the feedback of CM2 in two ways (CMs and its CH). When the CM transmits data, all other CMs in the cluster are listening. Each CM can hear the transmission of all other CMs within its broadcast range, and these CMs are generally neighbor nodes. The neighbor nodes of CM1 (including CM3, CM4, and CM5) will send their feedback to CM1 (including $F_{CM_{3},CM_{2}}\left(\Delta t\right)$, $F_{CM_{4},CM_{2}}\left(\Delta t\right)$, and $F_{CM_{5},CM_{2}}\left(\Delta t\right)$). The CH1 will send its feedback $F_{CH_{1},CM_{1}}\left(\Delta t\right)$ to CM1. Then, integrating its direct trust $D_{CM_{1},CM_{2}}\left(\Delta t\right)$, CM1 can obtain an overall trust $T_{CM_{1},CM_{2}}\left(\Delta t\right)$ for CM2 based on a fusion calculation method. CM-to-CM direct trust, CM-to-CM feedback, and CH-to-CM feedback constitute a cross-validation relationship.

**Figure 4:**
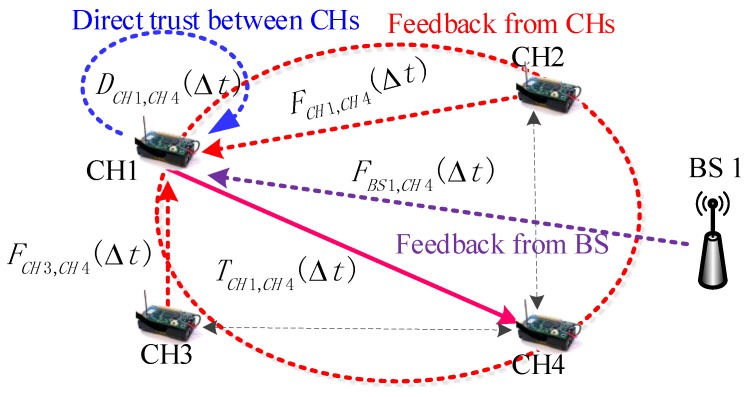
CH-to-CH overall trust computing based on the cross-validation mechanism. CH-to-CH direct trust, CH-to-CH feedback, and BS-to-CH feedback also constitute a cross-validation relationship.

A similar example of CH-to-CH overall trust computing based on the cross-validation mechanism is depicted in Figure 4. We can easily understand how to compute the overall trust in the CH-to-CH case from the CM-to-CM overall trust example in Figure 3. In this case, if CH1 needs to compute the overall trust of CH4 (CH1-to-CH4 overall trust $T_{CH_{1},CH_{4}}\left(\Delta t\right)$), CH1 will ask for the feedback of CM4 in two ways (CHs and its BS). In the case of Figure 4, CH2 and CH3 provide their CH-to-CH feedback $F_{CH_{2},CH_{4}}\left(\Delta t\right)$ and $F_{CH_{3},CH_{4}}\left(\Delta t\right)$ to CH1, and BS1 provides its BS-to-CH feedback $F_{BS_{1},CH_{4}}\left(\Delta t\right)$ to CH1. At the same time, CH1 needs to compute the direct trust of CH4 (CH1-to-CH4 direct trust $D_{CH_{1},CH_{4}}\left(\Delta t\right)$). After the collection of trust information, CH1 uses the theory of standard deviation analysis to perform the fusion calculation of overall trust $T_{CH_{1},CH_{4}}\left(\Delta t\right)$.

In the proposed FRAT scheme, evaluation methods are different for these trust (or feedback) relationships. Both $T_{CM_{x},CM_{y}}\left(\Delta t\right)$ and $T_{CH_{i},CH_{j}}\left(\Delta t\right)$ are trust decision credentials (or trust authorization credentials), and they can directly act as authorization credentials for node selection in data aggregation, fusion, and higher level transmission. However, $D_{CM_{x},CM_{y}}\left(\Delta t\right)$, $D_{CH_{i},CH_{j}}\left(\Delta t\right)$, $F_{CM_{x},CM_{y}}\left(\Delta t\right)$, $F_{CH_{i},CH_{j}}\left(\Delta t\right)$, $F_{CH_{i},CM_{x}}\left(\Delta t\right)$, and $F_{BS_{z},CH_{j}}\left(\Delta t\right)$ are trust evaluation factors. Each of these factors is one-sided and cannot fully reflect the interactive relationship of nodes in the entire system. Therefore, these factors cannot act as the authorization credential directly. We need to perform fusion calculations on these trust factors in order to obtain a more adequate and accurate overall trust. As mentioned in Section 1, in terms of accuracy, calculation speed, storage overhead, and communication overhead, the trust mechanism should be fast and resource-saving in order to provide services for a large number of resource-constrained nodes. In this work, we propose a series of fast and resource-saving trust computing methods for cooperation between CMs or between CHs. The calculation methods for these nodes’ trust (or feedback) relationships are introduced in Section 4.

The content of the feedback mainly includes three types of trust, that is the CM-to-CM overall trust degree, or CH-to-CM feedback trust, or CH-to-CH overall trust degree. In Section 4.1 and Section 4.2, we introduce the calculation methods of these three types of trust. According to the calculation methods in Section 4.1 and Section 4.2, the information transmitted during feedback should be a positive integer between one to 10.

### 3.3. Attack Pattern Analysis in the FRAT Scheme

In a clustered WSN, the ultimate goal of a trust system is to obtain accurate and reliable functionality against selfish or collaborative network attacks [10]. An effective trust computing system should have a good defense against malicious attacks, that is it should be able to resist selfish or cooperative attacks from the CH and CM. In a clustered WSN environment, network attacks may originate from both malicious CHs and CMs [41].

**Definition** **6.** ***Collaborative attacks from CMs or CHs.** As long as feedback is considered, a malicious CM or CH will provide dishonest feedback to structure a good CM or CH and/or increase the trust of its stakeholders. This type of attack is called a collaborative attack and is the most direct type of attack in a clustered WSN environment.*

The feedback from the cooperative nodes may produce incorrect trust evaluation results and how to adopt a defense mechanism to prevent cooperative attacks by malicious nodes is the key task of this work. After determining the attack methods of malicious nodes, we can create an effective trust calculation method to prevent malicious entities from achieving their goals by evaluating the behavior of malicious entities, thereby resisting such attacks. However, directly identifying collaborative attacks is a daunting task. In this study, we adopt an adaptive fusion computing method to eliminate false feedback based on the theory of bias analysis, in which the three trust factors are further cross-validated with each other. Compared with the traditional method, our mechanism based on the theory of bias analysis is a cross-validation trust computing mechanism. Compared with the other two values, the biased feedback is eliminated from the entire trust aggregation process.

## 4. Trust and Feedback Calculation in FRAT

As shown in Figure 2, Figure 3 and Figure 4, there are two types of direct trust relationship and four types of indirect feedback relationship in the clustered WSN environment. These trust factors have different computing systems because their attributes are completely different. In this section, we introduce related computing mechanisms for these trust factors.

### 4.1. CM-to-CM Overall Trust Calculation

**Figure 5:**
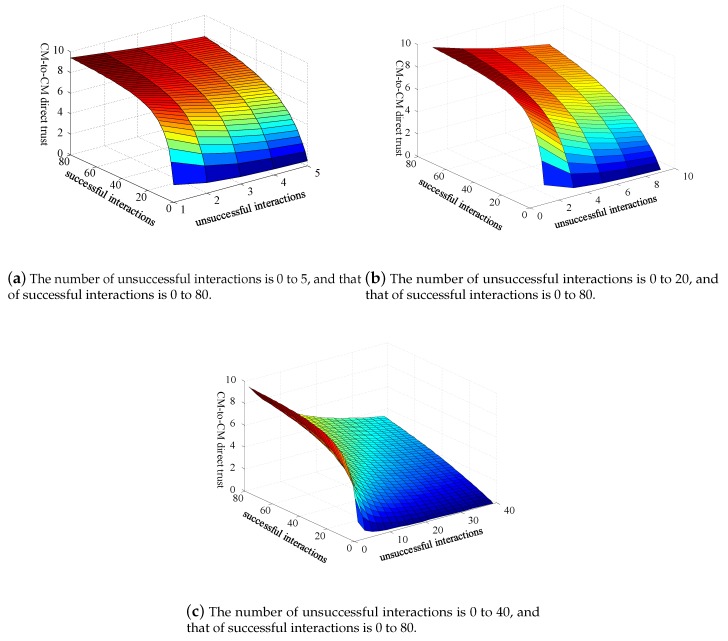
The value of CM-to-CM direct trust with penalty factor α=4.

**CM-to-CM direct trust calculation.** As mentioned earlier, the problem of saving overhead is the most basic WSN that requires resource constraints. In probability theory and statistics, the beta distribution is a series of continuous probability distributions defined on the interval [0, 1]. It is parameterized by two positive shape parameters, which are indexed by random variables. The form appears and controls the shape of the distribution. In [42], a beta trust system based on statistical theory was proposed. The system had the characteristics of flexibility and high resource efficiency. Inspired by the innovative work in [10,42], we used an improved betaprobability density function to calculate the CM’s direct trust in the CM. The direct trust calculation on the CM is defined by the following formula:

(1) $$\begin{array}{c} D_{CM_{x},CM_{y}}\left(\Delta t\right)\;\;\;\;\;\;\;\;\\ =\left\lceil 10\times E(\varphi \left(p|S_{CM_{x},CM_{y}}\left(\Delta t\right),U_{CM_{x},CM_{y}}\left(\Delta t\right)\right)\rceil \right. \end{array}$$

where Δt is a time window. The length Δt can be shorter or longer depending on the network analysis scheme. Therefore, as time goes by, the window forgets the old experience, but adds new experiences. The operation ⌈·⌉ is the closest integer function, such that ⌈0.82148⌉=8. The symbol *p* reflects the posterior probability of the binary event ($S_{CM_{x},CM_{y}}\left(\Delta t\right)$, $U_{CM_{x},CM_{y}}\left(\Delta t\right)$), and $S_{CM_{x},CM_{y}}\left(\Delta t\right)$ is the total number of successful data communications between nodes $CM_{x}$ with $CM_{y}$ during time Δt. $U_{CM_{x},CM_{y}}\left(\Delta t\right)$ is the total number of unsuccessful data communications between nodes $CM_{x}$ with $CM_{y}$ during time Δt. $E(\varphi \left(p|S_{CM_{x},CM_{y}}\left(\Delta t\right),U_{CM_{x},CM_{y}}\left(\Delta t\right)\right))$ is the expected probability of the beta distribution $\varphi (p|S_{CM_{x},CM_{y}}\left(\Delta t\right),U_{CM_{x},CM_{y}}\left(\Delta t\right))$:

(2) $$\begin{array}{c} \left\lceil E(\varphi \left(p|S_{CM_{x},CM_{y}}\left(\Delta t\right),U_{CM_{x},CM_{y}}\left(\Delta t\right)\right\rceil \right.\;\;\;\\ =\left\lceil \frac{10\times (S_{CM_{x},CM_{y}}\left(\Delta t\right)+1)}{S_{CM_{x},CM_{y}}\left(\Delta t\right)+\alpha *U_{CM_{x},CM_{y}}\left(\Delta t\right)+2}\right\rceil \end{array}$$

where positive integer α∈[1−N] is a punitive factor that reflects the punitive nature of failed interactions. In special cases, if $S_{CM_{x},CM_{y}}\left(\Delta t\right)+U_{CM_{x},CM_{y}}\left(\Delta t\right)=0$, which denotes no interactions between node $CM_{x}$ with $CM_{y}$ during time Δt. According to Equation (1), the value of $D_{CM_{x},CM_{y}}\left(\Delta t\right)=5$. If $S_{CM_{x},CM_{y}}\left(\Delta t\right)\neq 0$ and $U_{CM_{x},CM_{y}}\left(\Delta t\right)=0$, then the value of $D_{CM_{x},CM_{y}}\left(\Delta t\right)$ is a positive increasing value with the increase in the number of successful interactions. Figure 5 depicts the evolution trend of CM-to-CM direct trust. We can observe that the value of CM-to-CM direct trust quickly reduces with the increase in the number of failed interactions, which reflects the strictly punitive nature of the proposed trust mechanism for the failure of interactions.

**Figure 6:**
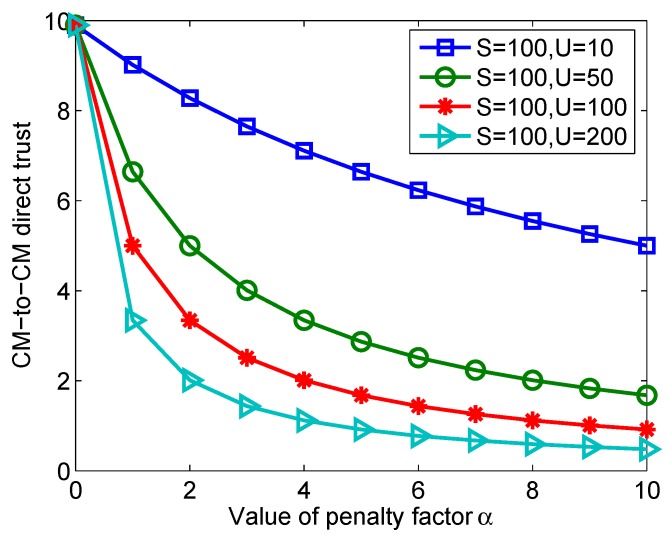
Analysis of CM-to-CM direct trust under different values of α.

Compared with the original method proposed by [42], the main difference of the improved beta probability density function is the penalty factor α to be introduced. If α=1, then our approach falls back to [42]. In α>1, then our approach reflects the punitive nature of the failure of interaction. We use Figure 6 for quantitative analysis of CM-to-CM direct trust under different values of penalty factor α. In Figure 6, *S* is the number of successful interactions, and *U* is the number of unsuccessful interactions. From Figure 6, the value of CM-to-CM direct trust shows a downward trend with increasing α, which reaches our design goal for punishment of failed interactions. In WSN systems with high security requirements, we should choose the value of α to approach 10.

**CM-to-CM feedback calculation.** As mentioned earlier, feedback is an important task for both CMs and CHs. It also provides information and key performance indicators for trust assessment. There are many collaborative CMs in a clustered WSN environment, and the feedback from these CMs is considered as a social rating and should have a high reference value for node trust evaluation. We used the improved beta probability density function with a strict punitive nature to compute $F_{CM_{x},CM_{y}}\left(\Delta t\right)$. As a result, the calculation efficiency was improved.

(3) $$F_{CM_{x},CM_{y}}\left(\Delta t\right)=\left\lceil \frac{10*(\xi \left(CM_{y}\right)+1)}{\left(\xi \left(CM_{y}\right)+\alpha *\gamma \left(CM_{y}\right)+2\right)}\right\rceil$$

where the positive integer α∈[1−N] is a penalty factor, which reflects the penalty nature for malicious feedback. $\xi (CM_{y})$ is the number of positive feedbacks (>0.5) toward $CM_{y}$ from other CMs in the cluster, whereas $\gamma (CM_{y})$ is the number of negative feedbacks (<0.5) from other CMs.

**CH-to-CM feedback calculation.** As shown in Figure 3, different from CM-to-CM feedback, the CH-to-CM feedback is a value based on the CH rating. We assumed that *I* CMs existed in a cluster. The CH would broadcast request packets in the cluster periodically. In response, all CMs in the cluster would forward their direct trust values to other CMs to the CH. CHs would then maintain these trust values in the matrix $f_{CH_{i}}$, as follows:

(4) $$f_{CH_{i}}=\left(\begin{array}{cccc} D_{CM_{1},CM_{1}} & D_{CM_{1},CM_{2}} & \cdots & D_{CM_{1},CM_{I}}\\ D_{CM_{2},CM_{1}} & D_{CM_{2},CM_{2}} & \cdots & D_{CM_{2},CM_{I}}\\ \; & \; & \ddots \\ D_{CM_{I},CM_{1}} & D_{CM_{I},CM_{2}} & \cdots & D_{CM_{I},CM_{I}} \end{array}\right)$$

where $D_{CM_{i},CM_{y}}$(i∈[1,I],y∈[1,I]) is the direct trust of a network member $CM_{i}$ for $CM_{y}$. In addition, if i=y, this means that the value is the node’s feedback for itself. In this study, an improved beta probability density function is used to calculate $F_{CH_{i},CM_{y}}\left(\Delta t\right)$.

(5) $$F_{CH_{i},CM_{x}}\left(\Delta t\right)=\left\lceil \frac{10*(g\left(CM_{y}\right)+1)}{\left(g\left(CM_{y}\right)+\alpha *b\left(CM_{y}\right)+2\right)}\right\rceil$$

where positive integer α∈[1−N] is the penalty factor, which reflects the penalty function of malicious feedback. $g(CM_{y})$ is the number of positive feedbacks (>0.5) toward $CM_{y}$ from other CMs in the cluster, whereas $b(CM_{y})$ is the number of negative feedbacks (<0.5) from other CMs. Analyzing Equations (4) and (5), we find that both feedback aggregation mechanisms are resource-saving methods with simple formulas and are suitable for resource-constrained wireless sensor networks with large sensor nodes.

The feedback value is a positive integer between one and 10. Thus, we can define how a CH/CM detects that a received feedback is positive or negative. If the value is less than or equal to five, we consider this feedback to be negative. If the value is more than five, we consider this feedback to be positive.

**CM-to-CM overall trust aggregating calculation based on standard deviation analysis.** As indicated in Definition 1, the *CM-to-CM* overall trust is evaluated based on three factors: $D_{CM_{x},CM_{y}}\left(\Delta t\right)$, $F_{CM_{x},CM_{y}}\left(\Delta t\right)$, and $F_{CH_{i},CM_{y}}\left(\Delta t\right)$. Therefore, aggregating these trust factors into a single value in an unbiased manner is a challenging problem. In statistics, standard deviation analysis means the absolute difference between any number in a set and the mean of the set [39,40]. The basic idea of standard deviation analysis is (1) to eliminate the number with a larger deviation than the other numbers and (2) to calculate the average of the remaining numbers.

We suppose that μ(Δt) is the summation value of the three trust factors at time stamp Δt. $f_{max}\left(\Delta t\right)$ is the maximum value of the three trust factors at time stamp Δt. $f_{min}\left(\Delta t\right)$ is their minimum value at the same time stamp Δt. γ(Δt) is the average value of the three trust factors. Then, the standard deviation of the three trust factors is defined as follows:

(6) $$\begin{array}{c} \delta \left(\Delta t\right)=\sqrt{\Omega (\Delta t)/3}\;\;\;,\\ \Omega \left(\Delta t\right)={\left(D_{CM_{x},CM_{y}}\left(\Delta t\right)-\gamma \left(\Delta t\right)\right)}^{2}+\;\;\;\\ {\left(F_{CM_{x},CM_{y}}\left(\Delta t\right)-\gamma \left(\Delta t\right)\right)}^{2}+\\ {\left(F_{CH_{i},CM_{y}}\left(\Delta t\right)-\gamma \left(\Delta t\right)\right)}^{2} \end{array}$$

From a statistical perspective, the standard deviation of a dataset is a measure of the amount of deviation between the observations contained in the dataset. Relying on the theory of bias analysis, we adopted an aggregation method for the overall trust, which could overcome the limitations of the traditional trust computing system [39,40]. The traditional trust mechanism weighs the attributes of the trust manually or subjectively.

(7) $$T_{CM_{x},CM_{y}}\left(\Delta t\right)=\left\{\begin{array}{c} \frac{\mu \left(\Delta t\right)-f_{max}\left(\Delta t\right)}{2},f_{max}\left(\Delta t\right)>\left(\gamma \left(\Delta t\right)+\delta \left(\Delta t\right)\right)\\ \frac{\mu \left(\Delta t\right)-f_{min}\left(\Delta t\right)}{2},f_{min}\left(\Delta t\right)<\left(\gamma \left(\Delta t\right)-\delta \left(\Delta t\right)\right)\\ \gamma (\Delta t)\;\;,otherwise\;\; \end{array}\right.$$

Compared with the traditional methods, our mechanism in Equation (7) performs adaptive trust calculation. The trust factor with a larger deviation compared with the other two values is eliminated from the overall trust aggregation process using Equations (5), (6), and (7). This removal solves the adaptive aggregation problem caused by collaborative attack CMs.

### 4.2. CH-to-CH Overall Trust Calculation

**CH-to-CH direct trust calculation.** We used a similar mechanism to calculate the direct trust from CH-to-CH, that is the direct trust from CM-to-CM. The direct trust assessment method on CHs is defined by the following formula:

(8) $$D_{CH_{i},CH_{j}}\left(\Delta t\right)=\left\lceil \frac{10\times (\psi_{CH_{i},CH_{j}}\left(\Delta t\right)+1)}{\psi_{CH_{i},CH_{j}}\left(\Delta t\right)+\alpha *\beta_{CH_{i},CH_{j}}\left(\Delta t\right)+2}\right\rceil$$

where α∈[1−N] is a penalty factor. Δt is a window of time. $\psi_{CH_{i},CH_{j}}\left(\Delta t\right)$ is the total number of successful data forwards. $\beta_{CH_{i},CH_{j}}\left(\Delta t\right)$ is the total number of unsuccessful data forwards of node $CH_{i}$ with $CH_{j}$ during time Δt.

**CH-to-CH feedback calculation.** In this study, we use an improved beta probability density function to calculate $F_{CH_{i},CM_{j}}\left(\Delta t\right)$.

(9) $$F_{CH_{i},CM_{j}}\left(\Delta t\right)=\left\lceil \frac{10*(\varpi \left(CH_{j}\right)+1)}{\left(\varpi \left(CH_{j}\right)+\alpha *\theta \left(CH_{j}\right)+2\right)}\right\rceil$$

where α∈[1−N] is a penalty factor. $\varpi (CH_{j})$ is the number of positive ratings (>0.5) toward $CH_{j}$ from other CHs, whereas $\theta (CH_{j})$ is the number of negative ratings (<0.5) from other CHs.

**BS-to-CH feedback calculation.** As shown in Figure 4, the BS-to-CH feedback is a value based on the BS rating. We assumed the existence of *J* CHs that interacted with a BS. The BS periodically broadcast the request packet for feedback. In response, all CHs forwarded their direct trust values to other CHs to the BS. The BS then maintained these trust values in the matrix $c_{CH_{i}}$:

(10) $$h_{CH_{i}}=\left(\begin{array}{cccc} D_{CH_{1},CM_{1}} & D_{CH_{1},CM_{2}} & \cdots & D_{CH_{1},CM_{J}}\\ D_{CH_{2},CM_{2}} & D_{CH_{2},CM_{2}} & \cdots & D_{CH_{2},CM_{J}}\\ \; & \; & \ddots \\ D_{CH_{J},CM_{1}} & D_{CH_{J},CM_{2}} & \cdots & D_{CH_{J},CM_{J}} \end{array}\right)$$

where $D_{CH_{i},CH_{j}}$(i∈[1,J],j∈[1,J]) is the direct trust of node $CH_{i}$ for $CH_{j}$. In this study, we use an improved beta probability density function to calculate $F_{BS_{z},CH_{j}}\left(\Delta t\right)$.

(11) $$F_{BS_{z},CH_{j}}\left(\Delta t\right)=\left\lceil \frac{10*(o\left(CH_{j}\right)+1)}{\left(o\left(CH_{j}\right)+\alpha *p\left(CH_{j}\right)+2\right)}\right\rceil$$

where α∈[1−N] is the penalty factor. $o(CH_{j})$ is the number of positive ratings (>0.5) toward $CM_{y}$ from other CHs, whereas $p(CH_{j})$ is the number of negative ratings (<0.5) from other CHs.

**CH-to-CH overall trust aggregating calculation based on standard deviation analysis.** We used an aggregation method for overall trust based on deviation analysis theory, which could overcome the limitations of traditional trust computing mechanisms where trusted attributes were weighted manually or subjectively [39]. We supposed that v(Δt) was the summation value of the three trust factors ($D_{CH_{i},CH_{j}}\left(\Delta t\right)$, $F_{CH_{i},CH_{j}}\left(\Delta t\right)$, and $F_{BS_{z},CH_{j}}\left(\Delta t\right)$) at time stamp Δt. $s_{max}\left(\Delta t\right)$ is the maximum value; $s_{min}\left(\Delta t\right)$ is its minimum value; and ρ(Δt) is the average value. The standard deviation of the three trust factors is defined as follows:

(12) $$\begin{array}{c} \omega \left(\Delta t\right)=\sqrt{\Psi (\Delta t)/3}\;\;\;,\\ \Psi \left(\Delta t\right)={\left(D_{CH_{i},CH_{j}}\left(\Delta t\right)-\rho \left(\Delta t\right)\right)}^{2}+\;\;\;\\ {\left(F_{CH_{i},CH_{j}}\left(\Delta t\right)-\rho \left(\Delta t\right)\right)}^{2}+\\ {\left(F_{BS_{z},CH_{j}}\left(\Delta t\right)-\rho \left(\Delta t\right)\right)}^{2} \end{array}$$

Then, the overall trust degree based on deviation analysis is defined as follows:

(13) $$T_{CH_{i},CH_{j}}\left(\Delta t\right)=\left\{\begin{array}{c} \frac{v\left(\Delta t\right)-s_{max}\left(\Delta t\right)}{2},s_{max}\left(\Delta t\right)>\left(\rho \left(\Delta t\right)+\omega \left(\Delta t\right)\right)\\ \frac{v\left(\Delta t\right)-s_{min}\left(\Delta t\right)}{2},s_{min}\left(\Delta t\right)<\left(\rho \left(\Delta t\right)-\omega \left(\Delta t\right)\right)\\ \rho (\Delta t)\;\;,otherwise\;\; \end{array}\right.$$

This trust aggregation in Equation (13) is an adaptive trust calculation mechanism. The trust factor with a larger deviation compared with the other two values is eliminated from the overall trust aggregation process using Equations (12) and (13).

## 5. Performance Analysis

In this section, we analyze the proposed trust mechanism from three aspects: (1) the attacker’s ability to resist collaborative attacks and the trust computing scheme itself, (2) time complexity, and (3) communication overhead (the latter two can reflect the computing speed and resource efficiency of the trust computing solution).

### 5.1. Time Complexity Analysis

We took some resource-saving steps to calculate the trust value between nodes, which was suitable for WSNs because it helped to save resources. In addition, we used an improved beta probability density function to calculate the overall trust value. It was found that this mechanism was a method to save resources and was suitable for resource-constrained nodes in large-scale sensor networks. Because the calculation of all these trust factors was a statistical operation, the computational overhead of the calculation could be ignored.

**Theorem** **1.** *Using the proposed trust evaluation scheme, the total time complexity of CM-to-CM overall trust computing was no more than O(g)+O(m)+O(k)+O(1).*

**Proof.**  In the period of CM-to-CM direct trust calculation (from Equation (1) to Equation (2)), the time complexity is O(g), and $g=S_{CM_{x},CM_{y}}\left(\Delta t\right)$+$U_{CM_{x},CM_{y}}\left(\Delta t\right)$. In the period of CM-to-CM feedback calculation (Equation (3)), the time complexity is O(m) and $m=\xi \left(CM_{y}\right)+\gamma \left(CM_{y}\right)$. In the period of CH-to-CM feedback calculation (from Equation (4) to Equation (5)), the time complexity is O(k) and $k=g\left(CM_{y}\right)+b\left(CM_{y}\right)$. In the period of CM-to-CM overall trust aggregating calculation (from Equation (6) to Equation (7)), the time complexity is O(1). Thus, the time complexity is O(g)+O(m)+O(k)+O(1). □

**Theorem** **2.** *Based on the proposed trust evaluation scheme, the total time complexity of CH-to-CH overall trust computing was no more than O(q)+O(w)+O(r)+O(1).*

**Proof.**  In the period of CH-to-CH direct trust calculation (Equation (8)), the time complexity is O(q) and $q=\psi_{CH_{i},CH_{j}}\left(\Delta t\right)+\beta_{CH_{i},CH_{j}}\left(\Delta t\right)$. In the period of CH-to-CH feedback calculation (Equation (9)), the time complexity is O(w) and $w=\varpi \left(CH_{j}\right)+\theta \left(CH_{j}\right)$. In the period of CS-to-CH feedback calculation (from Equation (10) to Equation (11)), the time complexity is O(r) and $r=o\left(CH_{j}\right)+p\left(CH_{j}\right)$. In the period of CH-to-CH overall trust aggregating calculation (from Equation (12) to Equation (13)), the time complexity is O(1). Thus, the time complexity is O(q)+O(w)+O(r)+O(1). □

In the period of trust factor measurement based on improved beta probability density functions (from Equation (1) to Equation (13)), the computing time complexity was no more than O(g)+O(m)+O(k)+O(1) (or O(q)+O(w)+O(r)+O(1)), which showed that the computing complexity of the proposed trust computing scheme was far superior to those of some existing schemes, such as the fuzzy-based trust models [11]), whose time complexity was $O(n^{3}log_{2}n)$. In traditional trust computing schemes, if n→∞, trust aggregation calculations would become extremely slow. In this study, we used a time-saving computer system that greatly increased the speed of trust calculation, which made the trust calculation scheme very suitable for large WSNs.

### 5.2. Communication Overhead Analysis

In order to analyze the communication overhead of the FRAT mechanism under full load conditions, we assumed that in the worst case, each CM wanted to communicate with other CMs in the cluster and each CH wanted to communicate with other CHs in the cluster. In addition, each CH needed to collect feedback from other CMs, and the BS must collect feedback reports from other CHs.

**Theorem** **3.** *Supposed that the network consists of J clusters and that the average size of clusters is I (including the CH of the cluster). Based on the proposed trust computing scheme, the maximum communication overhead is: $2I^{2}+2J^{2}+2I*J$.*

**Proof.**  (1) From Figure 3, in the cross-validation-based CM-to-CM overall trust calculations, feedback came from three sources. First, when node $CM_{i}$ wanted to collect feedback from node $CM_{x}$, the node $CM_{i}$ sent at most one CM feedback request, and this node $CM_{i}$ received a response. Second, $CM_{i}$ sent a feedback request to its CH and obtained feedback from the CH. Finally, $CM_{i}$ used its self-feedback information, which required no communication overhead. Therefore, if node $CM_{i}$ wanted to collect feedback from all nodes in the cluster, the maximum communication overhead became 2[(I−1)+1]=2I. If all nodes wanted to transfer data to each other, the maximum communication overhead was $2I*I=2I^{2}$.

(2) From Figure 3, in the cross-validation-based CH-to-CH overall trust calculation, the feedback came from three sources. First, when $CH_{j}$ wanted to collect feedback from $CH_{y}$, $CH_{j}$ sent a maximum of one CH feedback request, for which $CH_{i}$ received one response. Second, $CH_{j}$ sent one feedback request to its BS and received one feedback from the BS. Lastly, $CH_{j}$ used its self-feedback information, which did not require communication overhead. Therefore, if $CH_{j}$ wanted to collect feedback from all CHs in the network, the maximum communication overhead became 2[(J−1)+1]=2J. If all CHs wanted to communicate with one another, then the maximum communication overhead was $2J*J=2J^{2}$. In addition, in the trust calculation from CH to CH, when the CH wanted to collect feedback from its *I* members, it sent a *I* request and received a *I* response, thus resulting in a total communication overhead of 2I. Therefore, the overall trust of the largest communication overhead CH-to-CH was calculated as $2J^{2}+2I*J$. □

### 5.3. Anti-Collaborative Attack Ability Analysis

In Figure 2, Figure 3 and Figure 4, according to the inherent relationship among the three network entities, we propose the cross-validation mechanism, which is effective and reliable against collaborative attacks caused by malicious nodes. In this sub-section, we analyze the anti-collaborative attack ability of the FRAT scheme against collaborative attacks on the trust mechanism.

**Theorem** **4.** *Equations (1)–(3) consider not only the number of positive (or negative) ratings ($\xi (CM_{y})$ and $\gamma (CM_{y})$), but also the punitive nature for failed transactions. The feature of Equations (1)–(3) can effectively prevent collaborative attacks from accomplice CMs.*

**Proof.**  If $\gamma \left(CM_{y}\right)>\xi \left(CM_{y}\right)$, then $F_{CM_{x},CM_{y}}\left(\Delta t\right)\geq 5$, which covers a collaborative scenario where individual CMs attempt to lie about a bad CM [10,18]. We must prove that when $\gamma \left(CM_{y}\right)>\xi \left(CM_{y}\right)$, then $F_{CM_{x},CM_{y}}\left(\Delta t\right)<5$. From Equation (3), feedback from CMs can be calculated using the following improved beta probability density functions:

$$F_{CM_{x},CM_{y}}\left(\Delta t\right)=\left\lceil \frac{10*(\xi \left(CM_{y}\right)+1)}{\left(\xi \left(CM_{y}\right)+\alpha *\gamma \left(CM_{y}\right)+2\right)}\right\rceil ⟹$$

$$F_{CM_{x},CM_{y}}\left(\Delta t\right)\leq \frac{10*(\xi \left(CM_{y}\right)+1)}{\left(\xi \left(CM_{y}\right)+\alpha *\gamma \left(CM_{y}\right)+2\right)}$$

Under the case that $\gamma \left(CM_{y}\right)>\xi \left(CM_{y}\right)$, we must prove that $F_{CM_{x},CM_{y}}\left(\Delta t\right)<5$, that is,

$$\frac{\left(\xi \left(CM_{y}\right)+1\right)}{\left(\xi \left(CM_{y}\right)+\alpha *\gamma \left(CM_{y}\right)+2\right)}<\frac{1}{2}$$

Under the condition $\gamma \left(CM_{y}\right)>\xi \left(CM_{y}\right)$, a negative feedback exceeds a positive feedback. Thus, we only need to prove the following:

$$2\left(\xi \left(CM_{y}\right)+1\right)<\xi \left(CM_{y}\right)+\alpha *\gamma \left(CM_{y}\right)+2⟹$$

$$2\xi \left(CM_{y}\right)+2<\xi \left(CM_{y}\right)+\alpha *\gamma \left(CM_{y}\right)+2⟹$$

$$\xi \left(CM_{y}\right)<\alpha *\gamma \left(CM_{y}\right)$$

Due to $\gamma \left(CM_{y}\right)>\xi \left(CM_{y}\right)$ and α>1, $\xi \left(CM_{y}\right)<\alpha *\gamma \left(CM_{y}\right)$ must exist, which proves Theorem 4. □

Through Theorem 4, we proved that our trust system at the CM level had a protective ability against collaborative attacks from malicious nodes because this system could prevent such nodes from fulfilling their objectives.

**Theorem** **5.** *Equation (9) considers not only the number of positive (or negative) feedbacks from CHs, but also the punitive nature for failed transactions. The feature of Equation (7) can effectively prevent collaborative attacks caused by accomplice CHs.*

**Proof.**  We assumed that $\varpi (CH_{j})$ was the number of positive feedbacks and $\theta (CH_{j}$) was the number of negative feedbacks in CH-to-CH trust computing. If $\varpi \left(CH_{j}\right)<\theta \left(CH_{j}\right)$, then $F_{CH_{i},CH_{j}}\left(\Delta t\right)\geq 5$, which covers a collaborative attack scenario where individual CHs attempt to lie about a bad CH. We must prove that when $\varpi \left(CH_{j}\right)<\theta (CH_{j}$, then $F_{CH_{i},CH_{j}}\left(\Delta t\right)<5$. From Equation (9), feedback from CHs can be calculated using the following improved beta probability density functions:

$$F_{CH_{i},CM_{j}}\left(\Delta t\right)=\left\lceil \frac{10*(\varpi \left(CH_{j}\right)+1)}{\left(\varpi \left(CH_{j}\right)+\alpha *\theta \left(CH_{j}\right)+2\right)}\right\rceil ⟹$$

$$F_{CH_{i},CM_{j}}\left(\Delta t\right)\leq \frac{10*(\varpi \left(CH_{j}\right)+1)}{\left(\varpi \left(CH_{j}\right)+\alpha *\theta \left(CH_{j}\right)+2\right)}$$

We must prove that $F_{CH_{i},CM_{j}}\left(\Delta t\right)<5$, that is,

$$\frac{\left(\varpi \left(CH_{j}\right)+1\right)}{\left(\varpi \left(CH_{j}\right)+\alpha *\theta \left(CH_{j}\right)+2\right)}<\frac{1}{2}$$

Under the condition $\varpi \left(CH_{j}\right)<\theta \left(CH_{j}\right)$, the number of negative feedbacks exceeds the number of positive feedbacks. Thus, we only need to prove the following:

$$2\left(\varpi \left(CH_{j}\right)+1\right)<\varpi \left(CH_{j}\right)+\alpha *\theta \left(CH_{j}\right)+2⟹$$

$$2\varpi \left(CH_{j}\right)+2<\varpi \left(CH_{j}\right)+\alpha *\theta \left(CH_{j}\right)+2⟹$$

$$\varpi \left(CH_{j}\right)<\alpha *\theta \left(CH_{j}\right)$$

Based on known conditions, existing $\theta \left(CH_{j}\right)>\varpi \left(CH_{j}\right)$, and α>1, thus $\varpi \left(CH_{j}\right)<\alpha *\theta \left(CH_{j}\right)$ must be established, which proves Theorem 5. □

Through Theorem 5, we proved that our trust system at the CH level had a protective ability against collaborative attacks from malicious nodes because this system could prevent such nodes from fulfilling their objectives.

## 6. Experiment-Based Analysis and Evaluation

In this section, we first describe how to set up the experimental method in a simulated WSN environment, including how to deploy the recommended trust scheme on the simulated environment and how to set up the experimental configuration. The experimental results are then reported.

### 6.1. Experimental Methods and Parameters

Extensive experiments were conducted by using the NetLogo event simulator [10,43,44,45] to validate the effectiveness of FRAT. For comparison, we also added GTMS [18] and ATRM [36] into the simulator because both of them are independent of any specific routing mechanism.

In order to make the experiment closer to the real WSN environment, three types of nodes were deployed in the simulator according to their identity, namely the CM, CH, and BS [10]. The CM could be one of two types: good CM (GCM) and bad CM (BCM). The GCM always provided successful cooperation, while the BCM provided unsuccessful cooperation. The behavior of a CM as a feedback provider could be one of two types: honest CM (HCM) and malicious CM (MCM). The HCM always provided correct feedback to any CM, while the MCM always provided feedback to other CMs contrary to actual data. Similar to the CM, the GCH always provided successful cooperation, while the BCH provided unsuccessful cooperation. The HCH always provided the correct feedback, while the MCH always provided the opposite feedback of the actual data of the other CHs.

In the proposed trust computing scheme based on the cross-validation mechanism, the main threat was caused by malicious feedback. We designed several performance mechanisms for a comprehensive trust assessment scheme. Due to the limitation of the paper length, we mainly evaluated the performance of FRAT based on the following two aspects: the successful packet transmission rate under different MCMs and the successful packet transmission rate under different MCHs.

Table 2 lists the simulation parameters and default values used in the experiment. A total of 1000–10,000 nodes were deployed in the simulator, and an average of 100 CMs were deployed in each cluster. The penalty factor α was set at two to reflect a double punitive factor for selfish nodes or failed collaborators. The total time step of the simulation run was 1000, and the time window for trust calculation was 20. The percentage of the HCM was 30–100%, and the percentage of the HCH was 50–100%.

### 6.2. Evaluation under Different MCMs

We computed the packet successful delivery ratio (PSDR) [10] to reflect the reliability of the trust computing systems. A higher PSDR indicated higher reliability. In this set of experiments, we assumed that most CHs in the WSN environment were trusted, of which MCHs only accounted for 10%. This WSN environment was very similar to the actual situation, and most CHs were honest and trustworthy.

**Figure 7:**
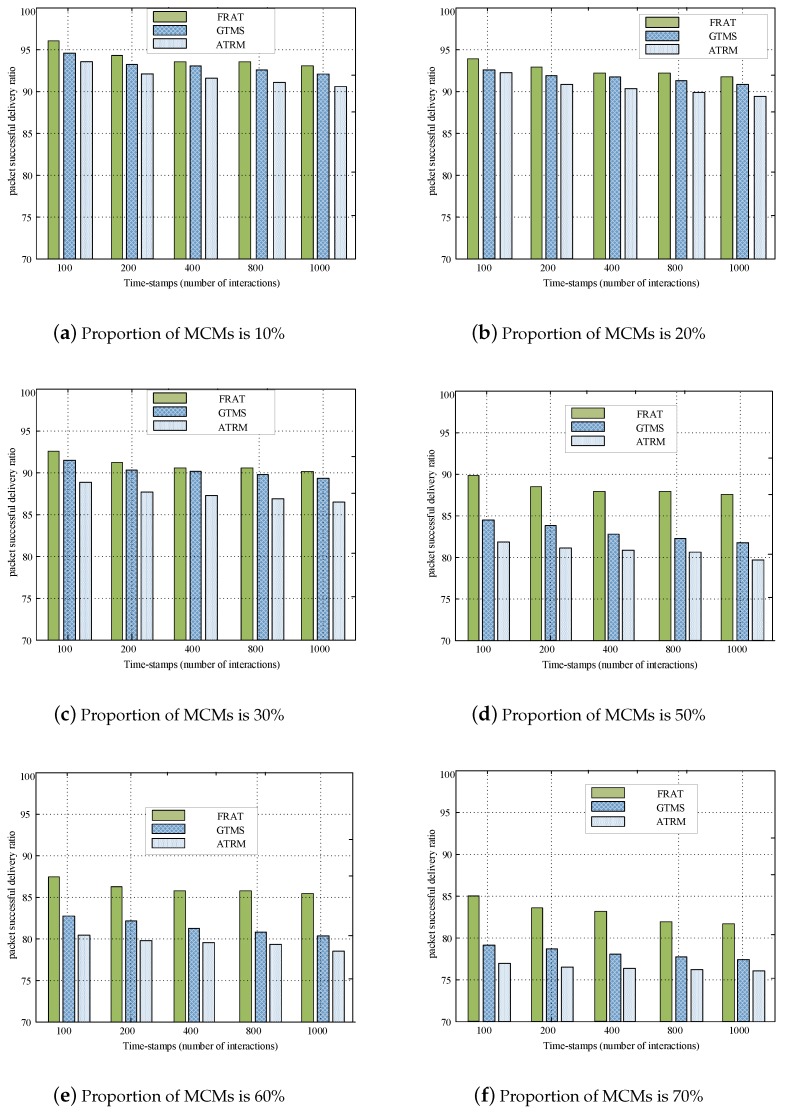
PSDR comparison with different percentages of malicious CMs (MCMs), where the proportion of malicious CHs is 10% GTMS, group-based trust computing mechanism; ATRM, a trust and reputation scheme.

Figure 7 illustrates the PSDR comparison at different percentages of the MCM. In this set of experiments, we assumed that the WSN environment was a trusted network community, of which 90% of the CHs were honest. The remaining 10% of CHs were malicious feedback providers. We set the percentage of MCMs to 10%, 20%, 30%, 50%, 60%, and 70%, which indicated that the cluster environment was fully honest (10%), honest (20%), relatively honest (30%), partly dishonest (50%), dishonest (60%), and fully dishonest (70%), respectively. Figure 7a shows a fully honest WSN environment, where the percentage of MCMs was only 10%. All three kinds of trust mechanisms had high PSDR values beyond 92%. These results reflected that the three kinds of trust mechanisms exhibited high reliability under an honest WSN community.

A robust trust mechanism should have a strong ability to counteract malicious behavior from MCMs. To evaluate the performance of the trust system under a more complex network environment, we gradually increased the proportion of malicious nodes. In Figure 7b–f, the proportion of MCMs were set to 20%, 30%, 50%, 60%, and 70%, and the results indicated larger differences compared with MCMs set to 10%. With the increase in the percentage of MCMs, the performance of GTMS and ATRM exhibited a marked decline; the PSDR of GTMS dropped to 93%, and the PSDR of ATRM dropped to 90%. The performance degradation may be mainly due to the usage of a one-way feedback mechanism. Relatively, FRAT exhibited robust performance in a complex network environment with a larger number of MCMs. These results were consistent with the actual situation, that is in a dishonest network environment, the MCM may conduct cooperative attacks, which may seriously affect the performance of the WSN environment. In order to improve the reliability of the proposed trust management mechanism, we adopted the idea that the overall trust of CM-to-CM was an adaptive combined value of bidirectional feedback (CM-to-CM feedback and CH-to-CH feedback). This new feedback mechanism could significantly improve the reliability of the proposed trust mechanism.

### 6.3. Evaluation under Different MCHs

**Figure 8:**
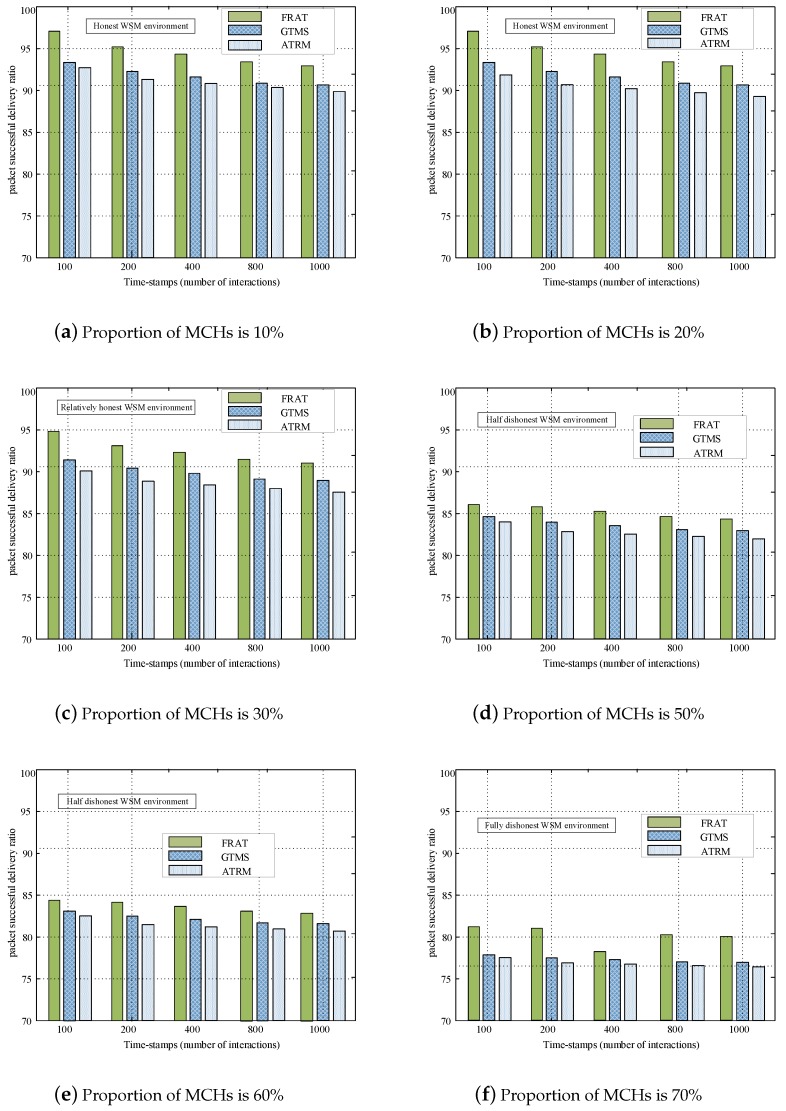
PSDR comparison with different percentages of MCHs, where the proportion of malicious CMs is 20%.

To evaluate the performance of the proposed trust mechanism at different MCH percentages, in this set of experiments, we assumed that each cluster environment was honest, and the MCM ratio was 20%. We set the proportion of MCHs to 10%, 20%, 30% 50%, 60%, and 70%, respectively. When the proportion of MCHs was set to 10%, the WSN environment was trustworthy. Most CHs in this network could keep their commitment and provide consistent stable feedbacks. When the proportion of MCHs was set to 20% or 30%, the WSN environment was relatively untrustworthy. More than half of the CHs in this WSN environment could keep their commitment and provide a consistently stable feedback. When the proportion of MCHs was set to 50%, the WSN environment was highly untrustworthy. Over half of the CHs in this WSN environment provided contrary feedback of the actual data for other CHs. Figure 8 shows a comparison of PSDR with different MCH percentages. A reliable trust computing system should have a strong ability to resist malicious behavior from MCHs.

In order to evaluate the performance of trust mechanisms in more complex network environments, we gradually increased the proportion of malicious CHs in the system, and the proportion of MCHs was set to 10%, 20%, 30%, 50%, 60%, and 70% in Figure 8a–f. Figure 8a shows an honest WSN environment, where the percentage of MCHs was only 10%. All three kinds of trust mechanisms had a high PSDR under this WSN environment, in which all values fluctuated around 90%. These results reflected that the three kinds of trust mechanisms exhibited high reliability under an honest WSN community.

With the increase in the percentage of MCHs, the WSN environment rapidly evolved from honest to fully dishonest. Figure 8d–f show that the performance of GTMS and ATRM exhibited a marked decline; the PSDR of GTMS dropped from 92% to 83%, and the PSDR of ATRM dropped from 90% to 82%. The performance degradation may be mainly due to the usage of a one-way feedback mechanism in GTMS and ARTM. Relatively, FRAT exhibited a more reliable performance in a complex network environment with a larger number of MCHs. These results were consistent with the actual situation, that is, in a dishonest network environment, MCHs may conduct cooperative attacks, which may seriously affect the performance of the WSN environment. To improve the reliability of the proposed trust management mechanism, we adopted the idea that the CH-to-CH overall trust was an adaptively merged value by the cross-validation feedback mechanism: CH-to-CH feedback and BS-to-CH feedback. This cross-validation feedback mechanism could significantly improve the anti-collaborative attack ability of the proposed trust mechanism. Thus, FRAT had a more robust reliability than GTMS and ATRM under five kinds of WSN environment, i.e., honest, relatively honest, partly dishonest, half dishonest, and fully dishonest, and it was suitable for trust computing under an open WSN.

### 6.4. Overhead Evaluation

**Figure 9:**
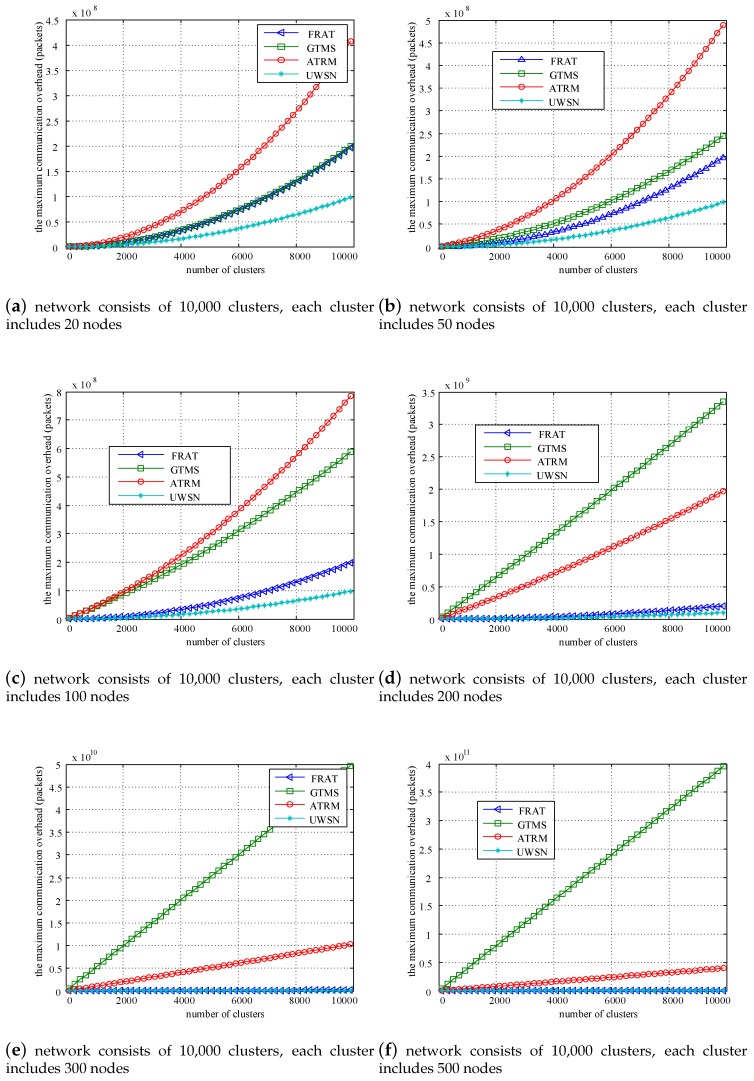
Comparing results of communication overhead under different network scales.

To evaluate the performance in a large-scale network environment, we adopted different cluster numbers and different cluster sizes. Figure 9 shows the compared results of communication overhead under different network scales. Six types of network environments were evaluated: (a) the network consisted of 10,000 clusters, and each cluster included 20 nodes; (b) the network consisted of 10,000 clusters, and each cluster included 50 nodes; (c) the network consisted of 10,000 clusters, and each cluster included 100 nodes; (d) the network consisted of 10,000 clusters, and each cluster included 200 nodes; (e) the network consisted of 10,000 clusters, and each cluster included 300 nodes; and (f) the network consisted of 10,000 clusters, and each cluster included 500 nodes. We compared our mechanism with GTMS [18], ATRM [36], and UWSN [20].

As the value of each feedback was a positive integer between one and 10, one byte was required for each feedback information. Table 3 lists the communication overhead (bytes) under full-load conditions. When the number of nodes in each cluster was relatively small (Figure 9a–c), we could observe that the communication overhead of FRAT was far below that of the other two trust mechanisms, GTMS and ATRM, but slightly larger than UWSN. The reason was that UWSN adopted a flat wireless sensor networks and did not require the overhead of the CH node. When the number of nodes in each cluster was relatively larger (Figure 9d–f), we could see that the communication overhead of FRAT was far below those of GTMS and ATRM. The communication overhead of FRAT gradually approached that of UWSN. According to Theorem 3. and Figure 9, the proposed trust computing scheme based on the cross-validation mechanism needed less communication overhead, and it was suitable for large-scale resource-constrained WSNs.

## 7. Conclusions

In this study, we proposed a trust computing scheme based on a cross-validation mechanism for clustered WSNs. Based on the theory of standard deviation analysis, this mechanism could remove the biased factor from multiple feedback sources. The theoretical analysis and experimental results provided useful insights. In a highly complex WSN environment with large percentages of malicious and selfish nodes, the proposed trust computing scheme based on the cross-validation mechanism may be insignificant, and thus, it should be given considerable attention in practical WSN applications. However, future work can pursue the following research directions:

- In a real deployment, nodes leave/join different clusters. Thus, future work can consider designing a scheme with node mobility.
- The proposed cross-validation mechanism was designed for clustered WSN. How to extend this mechanism to a flat WSN is another important direction.

## Figures and Tables

**Table 1:** The comparison of the trust computing mechanisms for WSNs. FRAT, fast, resource-saving, and anti-collaborative attack trust computing scheme.

Model	Clustered WSNs	Cross-Validation	Resource-Saving	Anti-Collaborative Attack	Trust Evaluation Approach
Desai model [12]	No	No	No	No	Subjective
Shaikh model [18]	Yes	No	Yes	No	Subjective
Raja model [34]	No	No	No	Yes	Subjective
Crosby model [37]	Yes	No	Yes	No	Subjective
Boukerche model [36]	No	No	Yes	No	Subjective
Zhan model [35]	No	No	No	No	Subjective
Li model [10]	Yes	No	Yes	No	Adaptive
FRAT in this paper	Yes	Yes	Yes	Yes	Adaptive

**Table 2:** Parameters and their possible values. HCM, honest CM; HCH, honest CH.

Symbol	Description	Possible Values
N=I×J	total number of CMs	1000–10,000
*I*	number of CMs in a cluster	100
*J*	total number of CHs	100–1000
*K*	total number of BSs	10
*t*	time-step of simulation runs	1000
Δt	time-window for trust computing	20
$H_{CM}$	percentage of HCMs	30–100%
$H_{CH}$	percentage of HCHs	50–100%
α	penalty factor	2

**Table 3:** Communication overhead under full-load conditions.

	Communication Overhead
FRAT	$2I^{2}+2J^{2}+2I*J$
GTMS	2J(I(I−1)(I−2)+(J−1)
ATRM	4J(I(I−1)+(J−1))
UWSN	$2J^{2}$
